# Functional peptide-mediated plastid transformation in tobacco, rice, and kenaf

**DOI:** 10.3389/fpls.2022.989310

**Published:** 2022-09-23

**Authors:** Masaki Odahara, Yoko Horii, Jun Itami, Kenta Watanabe, Keiji Numata

**Affiliations:** ^1^ Biomacromolecule Research Team, RIKEN Center for Sustainable Resource Science, Wako, Japan; ^2^ Department of Material Chemistry, Kyoto University, Kyoto, Japan

**Keywords:** plastid transformation, gene delivery, functional peptide, gene targeting, tobacco, rice, kenaf

## Abstract

In plant engineering, plastid transformation is more advantageous than nuclear transformation because it results in high levels of protein expression from multiple genome copies per cell and is unaffected by gene silencing. The common plastid transformation methods are biolistic bombardment that requires special instruments and PEG-mediated transformation that is only applicable to protoplast cells. Here, we aimed to establish a new plastid transformation method in tobacco, rice, and kenaf using a biocompatible fusion peptide as a carrier to deliver DNA into plastids. We used a fusion peptide, KH-AtOEP34, comprising a polycationic DNA-binding peptide (KH) and a plastid-targeting peptide (AtOEP34) to successfully deliver and integrate construct DNA into plastid DNA (ptDNA) *via* homologous recombination. We obtained transformants in each species using selection with spectinomycin/streptomycin and the corresponding resistance gene *aadA*. The constructs remained in ptDNA for several months after introduction even under non-selective condition. The transformants normally flowered and are fertile in most cases. The offspring of the transformants (the T_1_ generation) retained the integrated construct DNA in their ptDNA, as indicated by PCR and DNA blotting, and expressed GFP in plastids from the integrated construct DNA. In summary, we successfully used the fusion peptide method for integration of foreign DNA in tobacco, rice, and kenaf ptDNA, and the integrated DNA was transmitted to the next generations. Whereas optimization is necessary to obtain homoplasmic plastid transformants that enable stable heterologous expression of genes, the plastid transformation method shown here is a novel nanomaterial-based approach distinct from the conventional methods, and we propose that this easy method could be used to target a wide variety of plants.

## Introduction

Plastids are plant organelles that can transform their function and morphology depending on tissue type. For example, in leaves, plastids known as chloroplasts perform photosynthesis; in roots and storage tissues, amyloplasts accumulate starch; and in fruits and flowers, chromoplasts biosynthesize and store pigments. Plastids possess their own genomic DNA and gene expression system, distinct from those of the nucleus. Plastid genomic DNA (ptDNA) is generally mapped as a 100–200-kb circular structure comprising a large and a small-single copy region separated by a pair of large inverted repeats. ptDNA is essential as it encodes components of the photosynthetic pathway and genes for gene expression in plastids. The abundance of plastids in cells and multiple plastid genome copies enable high production of recombinant proteins in plastids—more than 75% of the total soluble protein (Castiglia et al., 2016). This protein production is unaffected by gene silencing, unlike the expression of exogenous genes in the nucleus. Furthermore, the maternal inheritance of chloroplast genes prevents the unintended spread of foreign genes by pollen. Therefore, plastids are often the target of genetic modification *via* genetic transformation for the production of valuable proteins (Bock, 2021). The involvement of plastids in the production of secondary metabolites also suggests that there is considerable potential for metabolic engineering of plastids (Jensen and Scharff, 2019).

The two most common methods of genetic modification in plastids are biolistic bombardment and polyethylene glycol (PEG)-mediated transformation. Biolistic bombardment utilizes submicrometer-sized metal particles coated with DNA; the particles are shot into plant tissues to deliver the DNA into plant cells. Plastid transformation *via* biolistic bombardment has been established in many plant species, including model plants, such as tobacco (*Nicotiana tabacum* (Svab et al., 1990) and Arabidopsis (*Arabidopsis thaliana*; (Ruf et al., 2019), and crop plants, such as tomato (*Solanum lycopersicum* (Ruf et al., 2001) and chickpea (*Glycine max*; (Dufourmantel et al., 2007). However, efficient plastid transformation methods are still lacking for many plant species. In particular, homoplasmic plastid transformation, which is the replacement of all copies of wild-type plastid genomes with modified plastid genomes, has not been established in many species. In rice, stable plastid transformation was achieved *via* long-term selection with tissue culture, but the transformants were sterile (Wang et al., 2018). Furthermore, it was recently discovered that off-target mutations of nuclear DNA can be induced during biolistic transformation (Liu et al., 2019), raising concern about nuclear off-target mutation even for plastid transformation. PEG-mediated plastid transformation, which introduces DNA into protoplast cells *via* the action of PEG, has been established in several plant species, including *N. tabacum* (Golds et al., 1993). However, this method requires regeneration from protoplasts to obtain transgenic plants, which limits the number of plants it can be applied to. In both biolistic bombardment and PEG-mediated transformation, spectinomycin (Sp) and streptomycin (Str), which inhibit translation by binding to ribosomes in plastids, are commonly used for selection in combination with the resistance marker gene aminoglycoside-3′′-adenylyltransferase, *aadA*, driven by a plastid endogenous promoter such as P*rrn*. In general, sequences homologous to the plastid DNA (homology arms; typically longer than 1 kb) are arranged at both sides of the marker to induce integration of the construct DNA into ptDNA by homologous recombination. Marker selection is essential for plastid transformation because a cell contains thousands of copies of ptDNA. Homoplasmic transformation can be achieved by selection from transformed cells, in which only a small amount of ptDNA is assumed to carry the integrated marker gene.

Functional peptides have been used to deliver biomacromolecules into cells because of their functional potency and high design flexibility. Fusion peptides are composed of a combination of functional peptides. Cell-penetrating peptides (CPPs) are short peptides that can pass through cell membranes (Di Pisa et al., 2015); polycationic peptides are short peptides composed of positively charged amino acids that bind to nucleic acids; and organelle-targeting peptides are endogenous signal peptides that sort proteins to organelles (Watanabe et al., 2021). Using fusion peptides as carriers, DNA (Lakshmanan et al., 2013; Lakshmanan et al., 2015; Midorikawa et al., 2019; Miyamoto et al., 2019), RNA (Numata et al., 2014; Thagun et al., 2020), and protein (Ng et al., 2016; Numata et al., 2016; Guo et al., 2019) can be successfully delivered into plant cells in a wide variety of tissue types. With organelle-targeting peptides and polycationic peptides, fusion peptides selectively deliver DNA into plant organelles, plastids and mitochondria (Chuah et al., 2015; Yoshizumi et al., 2018). For plastids, KH-AtOEP34, a fusion peptide composed of the cationic DNA-binding KH sequence and the plastid-localizing peptide of outer envelope membrane protein OEP34/TOC34 (Li and Chen, 1997; Li and Teng, 2013), was developed. This peptide enables selective delivery of plasmid DNA (pDNA) into plastids and expression of reporter genes from the pDNA (Yoshizumi et al., 2018; Thagun et al., 2019).

Here, we develop a peptide-mediated plastid transformation method using KH-AtOEP34 in combination with Sp/Str selection in a model plant (tobacco) and a crop plant (rice). We also tested the peptide-mediated plastid transformation in kenaf (*Hibiscus cannabinus*), a fiber-enriched crop plant for which no plastid transformation method previously existed. We report the stable integration of a construct DNA into the ptDNA of all three species by the fusion-peptide-mediated gene delivery method. The integrated exogeneous genes were successfully transmitted to the next generations of each plant.

## Materials and methods

### Plant materials and growth condition

Tobacco (*Nicotiana tabacum* ‘Petit Havana SR1’), rice (*Oryza sativa* ‘Nipponbare japonica’), and kenaf (*Hibiscus cannabinus*) (Fujita Seed Co., Japan) were used in this study. For tobacco, seeds were sterilized with 70% ethanol for 1 min followed by 0.5% sodium hypochlorite for 20 min and then washed with sterilized water. The seeds were cultivated on rooting medium [Murashige-Skoog (MS) medium (Murashige and Skoog, 1962) supplemented with 3% sucrose and 0.3% phytagel (Sigma-Aldrich, USA)] and then cultivated at 25°C under a 16 h light condition in a growth chamber. For flowering and seed maturation, tobacco plants were cultivated in a 2:1 mixture of soil and vermiculite at 28°C under a 14 h light condition.

For rice, peeled seeds were sterilized with 70% ethanol for 1 min followed by 0.5% sodium hypochlorite for 20 min with rotation and then washed with sterilized water. The sterilized seeds were cultivated on N6D medium [3% sucrose, 0.03% casamino acids, 0.4% Chu basal salt mix (Merck, Germany), 0.288% proline, 0.01% myo-inositol, 2 mg/L 2,4-D, 0.5 mg/L nicotinic acid, 0.5 mg/L pyridoxine HCl, 1 mg/L thiamine HCl, 2 mg/L glycine, 0.4% phytagel, pH 5.8] at 30°C under continuous light to induce calli. For flowering and seed maturation, rice plants were cultivated in nursery soil (Honens nursery soil No. 1, Honen Agri Co., Japan) at 30°C under a 14 h light condition.

For kenaf, seeds were sterilized with 70% ethanol for 20 min followed by 1% sodium hypochlorite for 30 min and then washed with sterilized water. Seeds were placed on a rooting medium [MS medium supplemented with 3% sucrose and 0.3% phytagel (Sigma-Aldrich, USA), pH 5.8] and then cultivated at 22°C under a 16 h light condition in a growth chamber. For callus induction, cotyledons of kenaf seedlings were cut and placed on a callus-inducing medium (CIM) [MS supplemented with 2% sucrose, 1.5 mg/L 6-benzylaminopurine (6-BAP), 0.01 mg/L indole-3-butyric acid (IBA), pH 5.8]. For flowering and seed maturation, kenaf plants were cultivated in a 2:1 mixture of soil and vermiculite at 22°C under an 8 h light condition.

### Plasmid construction

**Figure 1:**
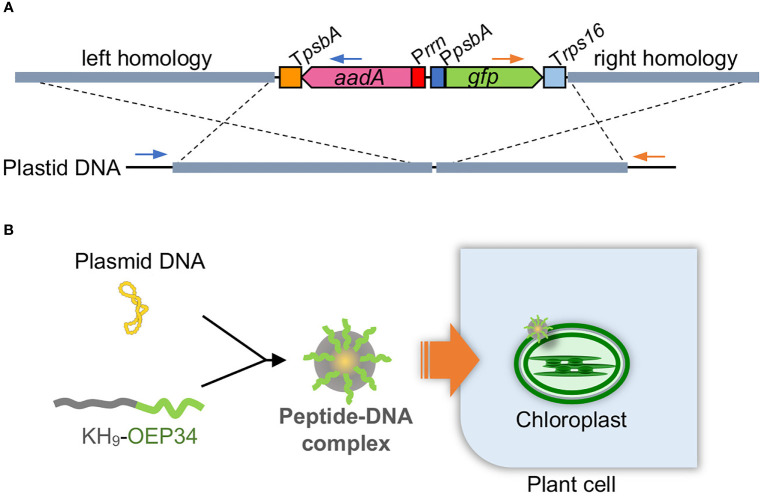
Peptide-mediated plastid transformation. **(A)** Construct DNA (above) targeting plastid DNA (below) by homologous recombination. A marker gene cassette (P*rrn*-*aadA*-T*psbA*) and a reporter gene cassette (P*psbA*-*gfp*-T*rps16*) are arranged between the left and right homology arms. Left and right homology arms are 1.8 and 1.2 kb for tobacco, 1.9 and 1.4 kb for rice, and 2.0 and 1.3 kb for kenaf. The construct DNA is integrated into plastid DNA *via* homologous recombination as shown by dashed cross lines. Primes used to test left and right integration are shown by blue and orange primers, respectively. **(B)** Scheme for peptide-mediated DNA delivery into plastids. The construct plasmid DNA is complexed with the fusion peptide KH_9_-OEP34 that enables delivery of the construct DNA into plastids.

Plasmids targeting plastids of each plant were constructed based on a tobacco plastid transformation plasmid, which is composed of 1.8-kb left and 1.2-kb right homology arms to target the *trnV*-*rps12/7* intergenic region (Zoubenko et al., 1994) and *aadA* and *gfp* gene expression cassettes in between the homology arms (**Figure 1A**).

For rice, the *trnV*-*rps12/7* region (Hiratsuka et al., 1989) amplified with primers P1 and P2 (primer sequences are listed in **Table S4**) was inserted into the BamHI site of pUC19. The *aadA* and *gfp* gene expression cassettes amplified with primers P3 and P4 from the tobacco plastid transformation plasmid were then inserted into the BglII site of the rice *trnV*-*rps12/7* region, resulting in 2.2-kb left and 1.1-kb right homology arms.

For kenaf, the *trnV*-*rps12/7* region amplified with primers P5 and P6 was inserted into pUC19 using an In-Fusion cloning kit (TaKaRa, Japan). The *aadA* and *gfp* gene expression cassettes amplified with primers P7 and P8 from the tobacco plastid transformation plasmid were then inserted into the BglII site of the kenaf *trnV*-*rps12/7* region using an In-Fusion cloning kit, resulting in 2.0-kb left and 1.3-kb right homology arms.

### Peptide-mediated plastid transformation

For tobacco plastid transformation, 10 μg of the targeting plasmid DNA was mixed with 3.2 μg of the fusion peptide KH-AtOEP34 [KHKHKHKHKHKHKHKHKHMFAFQYLLVM (28 a.a., 3650.4 Da)] in 1000 μL solution and then incubated at room temperature for 30 min. For DNA only treatment, same concentration of plasmid DNA solution was prepared without the peptide. Cut tobacco leaf pieces were submerged in the solution and then subjected to vacuum (-0.08 MPa) for 1 min followed by pressure (+0.08 MPa) for 1 min. After being washed with sterilized water, the leaf pieces were cultivated on MSBN [MS supplemented with 3% sucrose, 1 mg/L 6-BAP, 0.1 mg/L naphthaleneacetic acid (NAA), 0.3% phytagel, pH 5.8] for 4 d and then on MSBN medium containing 500 mg/L spectinomycin. Shoots regenerated from the leaf explants were subjected for additional two rounds of regeneration cycles to increase the ratio of the construct DNA-containing ptDNA. Finally, shoots were transferred to the rooting medium containing 500 mg/L spectinomycin.

For rice plastid transformation, 10 μg of the targeting plasmid DNA was mixed with 3 μg of the fusion peptide KH-AtOEP34 in 400 μL solution and then incubated at room temperature for 30 min. For DNA only treatment, same concentration of plasmid DNA solution was prepared without the peptide. Approximately 200 mg of calli induced from germinated seeds were submerged in the solution and then subjected to vacuum (-0.08 MPa) for 1 min followed by pressure (+0.08MPa) for 1 min. After cultivation on N6D medium for 5 d, the calli were cultivated on REIII medium [MS supplemented with 3% sucrose, 3% sorbitol, 0.4% casamino acid, 0.02 mg/L NAA, 2 mg/L kinetin, 100 mg/L myo-inositol, 0.5 mg/L nicotinic acid, 0.5 mg/L pyridoxine HCl, 0.1 mg/L thiamine HCl, 2 mg/L glycine, 0.4% phytagel, pH 5.8] containing 200 mg/L Str. Regenerated green shoots of the transformed rice were cultivated on a rooting medium [MS supplemented with 3% sucrose, 100 mg/L myo-inositol, 0.5 mg/L nicotinic acid, 0.5 mg/L pyridoxine HCl, 0.1 mg/L thiamine HCl, 2 mg/L glycine, 0.4% phytagel, pH 5.8] containing 300 mg/L Str. No additional round of regeneration was performed for rice plastid transformation.

For kenaf plastid transformation, 15 μg of the targeting plasmid DNA was mixed with 5 μg of the fusion peptide KH-AtOEP34 in 500 μL solution and then incubated at room temperature for 30 min. For DNA only treatment, same concentration of plasmid DNA solution was prepared without the peptide. Cut cotyledon pieces or calli were submerged in the solution and then subjected to vacuum (-0.08 MPa) for 1 min followed by pressure (+0.08 MPa) for 1 min. After being washed with sterilized water, the cotyledon pieces and calli were cultivated on CIM containing 100 mg/L Sp and then transferred to SIM containing 100 mg/L Sp. No additional round of regeneration was performed for kenaf plastid transformation.

### Genotyping of transformants

**Figure 2:**
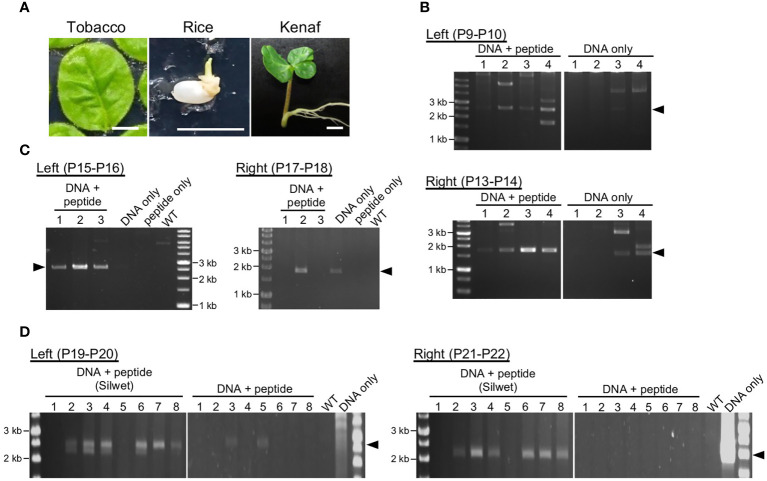
Transient integration of the construct DNA into plastid DNA. **(A)**. Plant materials used for DNA delivery: tobacco leaf, rice callus induced from seed, and kenaf cotyledon. Bars, 1 cm. **(B–D)**. Analysis of construct DNA integration into plastid DNA of tobacco **(B)**, rice **(C)**, and kenaf **(D)**. PCR genotyping of recombination between the construct DNA and the plastid DNA *via* left and right homology arms. Arrowheads denote positions of the predicted recombination products: 2.5 and 1.7 kb **(B)**, 2.6 and 1.9 kb **(C)**, and 2.5 and 2.4 kb **(D)** for the left and right arms, respectively. Genotyping was performed 11, 3, and 14 d after introduction for tobacco, rice, and kenaf, respectively. WT, wild-type plants without any treatment. For kenaf, each lane include PCR products from five cotyledon pieces. Primers used in the PCR are shown in each panel, and the details of the position of the primers are shown in **Figure S1**.

**Figure 3:**
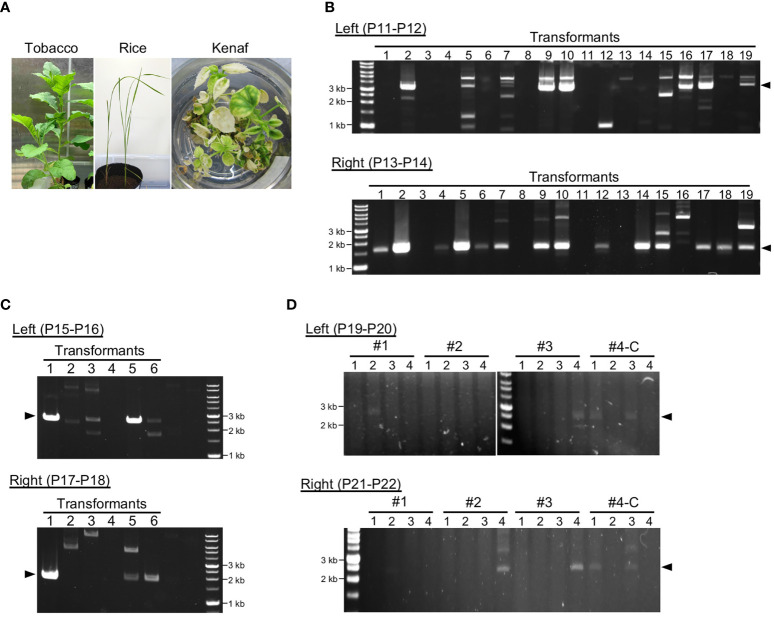
Genotyping of transformants after long-term cultivation. **(A)** Transformants of each plant after cultivation for 3–4 months. **(B–D)**. Analysis of construct DNA integration into plastid DNA of tobacco **(B)**, rice **(C)**, and kenaf **(D)**. PCR genotyping of recombination between the construct DNA and the plastid DNA *via* left and right homology arms. Arrowheads indicate the expected product sizes: 3.3 and 1.7 kb **(B)**, 2.6 and 1.9 kb **(C)**, and 2.5 and 2.4 kb **(D)** for the left and right arms, respectively. Genotyping PCR was performed 4, 3, and 5 months after construct introduction for tobacco, rice, and kenaf, respectively. For kenaf, #1–#4-C refer to individual transformants, and numbers under them refer to different leaves or shoots of the transformants (see **Figure S6**). #1–3 and #4-C were obtained using cotyledons and calli, respectively. Primers used in the PCR are shown in each panel, and the details of the position of the primers are shown in **Figure S1**.

**Figure 4:**
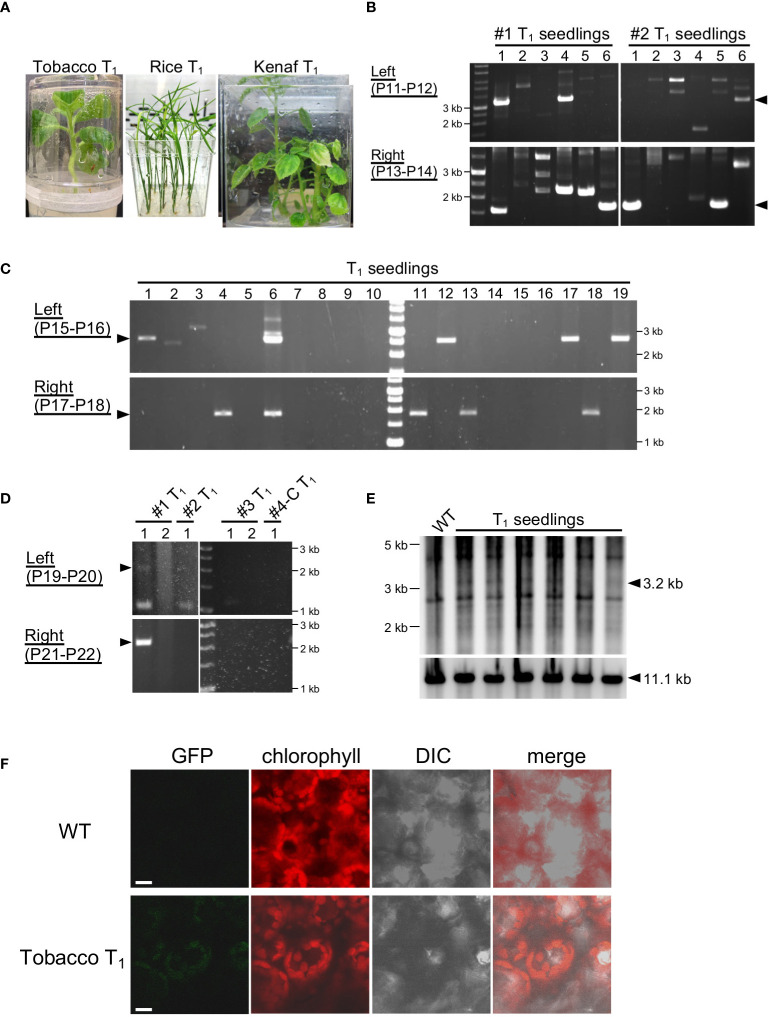
Genotyping of T_1_ generations. **(A)** T_1_ seedlings of tobacco, rice, and kenaf grown on selection medium. **(B–D)** PCR genotyping of T_1_ seedlings of tobacco **(B)**, rice **(C)**, and kenaf **(D)**. Arrowheads indicate the expected product size: 3.3 and 1.7 kb **(B)**, 2.6 and 1.9 kb **(C)**, and 2.5 and 2.4 kb **(D)** for the left and right arms, respectively. For kenaf, #1–#4-C refer to T_1_ of individual transformants, and numbers under them refer to different offspring of the transformants. Primers used in the PCR are shown in each panel, and the details of the position of the primers are shown in **Figure S1**. **(E)**. DNA gel blots of the plastid DNA of tobacco T_1_ seedlings. WT, wild type. **(F)**. Imaging of GFP in tobacco plastids. GFP fluorescence from leaves of tobacco T_1_ and WT seedlings were observed by CLSM. Chlorophyll autofluorescence denotes plastids. Bars, 10 μm.

PCR was used for genotyping of transformants using the primers listed in **Table S4**. For tobacco, the primers P9 and P10 (**Figure 2B**) or P11 and P12 (**Figures 3B** and **4B**) were used to determine integration in the left arm, and P13 and P14 were used to determine integration into the right arm. For rice, P15 and P16 were used to determine integration into the left arm and P17 and P18 were used to determine integration into the right arm. For kenaf, P19 and P20 were used to determine integration into the left arm and P21 and P22 were used to determine integration into the right arm. Positions of the genotyping primers are shown in **Figure S1**.

### DNA gel blotting

For gel blotting, genomic DNA was extracted using the cetyltrimethylammonium bromide (CTAB) method (Murray and Thompson, 1980), digested with SmaI, and electrophoresed in a 0.75% agarose gel. The genomic DNA was transferred to a nylon membrane and hybridized with a probe prepared with a DNA fragment amplified with primers P23 and P24 (**Table S4**) with tobacco genomic DNA using the alkPhos Direct Labelling System (Cytiva, US). The probe was detected using CDP-Star (Cytiva, US) and an Image Analyzer LAS-3000 (Fuji Film, Japan).

### Observation of GFP fluorescence

Leaves from 10-day-old tobacco T_1_ seedlings cultivated on MS supplemented with 3% sucrose, 0.3% phytagel, and 500 mg/L spectinomycin were observed by confocal laser scanning microscopy (TCS SP8; Leica Microsystems, Germany). To eliminate chlorophyll autofluorescence, GFP fluorescence was observed with gate-on time 0.3-1.2 according to Kodama (2016).

## Results and discussion

### Peptide-mediated plastid transformation

To target ptDNA *via* homologous recombination, we constructed plasmids composed of left and right homology arms, with a marker and reporter gene cassette between the homology arms (**Figure 1**). The left and right homology arms are 1.8–2.2- and 1.1–1.4-kb sequences homologous to ptDNA of each plant to target a locus between the 16S rDNA genes *trnV* and *rps12/7*, which has been used previously for integration of exogeneous DNA into ptDNA (Maliga and Tungsuchat-Huang, 2014; Daniell et al., 2016). The marker and reporter genes are *aadA*, which conveys resistance to spectinomycin/streptomycin, and *GFP* genes driven by the endogenous plastid promoters of *rrn* and *psbA*, respectively. We took care in choosing suitable tissues for peptide-mediated delivery of construct DNA, since the growth stage and tissue type of plant materials affect transformation efficiency in general (**Figure 2A**). For tobacco, we selected true leaves, as previously used in the biolistic bombardment method (Maliga and Tungsuchat-Huang, 2014). For rice, we used the peptide-mediated plastid transformation method on callus induced from germinated seeds, which is sufficient for peptide-mediated delivery of plasmid DNA (Miyamoto et al., 2020) and can regenerate shoots efficiently (Toki et al., 2006). For kenaf, we selected cotyledons, which can induce callus efficiently under appropriate hormone treatment (Odahara et al., 2020).

To deliver construct DNA to plastids, each construct DNA was complexed with KH-AtOEP34 at an N/P ratio (defined as molar ratio of cationic peptide nitrogen to anionic pDNA phosphate) of 0.5, which results in the highest introduction efficiency (Lakshmanan et al., 2015; Chuah and Numata, 2018). We submerged plant materials in the construct DNA–peptide complex solution and then subjected them successively to vacuum and pressure treatment to facilitate infiltration of the complex. At 3–14 d after infiltration, we evaluated transient integration of the construct DNA into ptDNA using genotyping PCR covering the junction region between the construct DNA and the ptDNA (**Figure S1**). Genotyping PCR of tobacco (**Figure 2B**) and rice (**Figure 2C**) showed amplification of products that corresponded to the size of the predicted products from the integrated ptDNA, associated with other multiple products. We confirmed the predicted products by sequencing (**Figure S2**) and interpreted other products to be derived from non-specific amplification as they were not reproducible or were also amplified from explants treated with DNA only. For tobacco, the numbers of explants with successful integration and the amount of the products suggest that integration of construct DNA into ptDNA *via* each homology sequence arm was more efficient in explants and calli treated with construct DNA and peptides than in those treated with construct DNA only. For kenaf, genotyping PCR showed inefficient integration of construct DNA into ptDNA without any pretreatment even for the explants treated with plasmid DNA and peptides (**Figure 2D**). However, pretreating kenaf cotyledons with the surfactant Silwet L-77 likely improved the integration efficiency as judged by the number of explants with successful integration and by the amount of the PCR products (**Figure 2D**), suggesting water-repellent of kanaf leaf should be alleviated by the treatment with Silwet L-77. These genotyping results indicate that the fusion peptide method resulted in efficient gene delivery and integration of construct DNA into the ptDNA of each plant.

To obtain stable transplastomic plants, we subjected explants or calli infiltrated with the pDNA–peptide complex to shoot regeneration under selection for Sp or Str resistance. For tobacco under Sp selection, 85 shoots were regenerated from ~7000 samples (**Figure S3**), and the regenerated shoots were subjected for additional rounds of regeneration to increase ptDNA containing construct DNA. Most of the tobacco shoots grew normally under the selection condition, and the plants were subsequently transferred to soil without any antibiotics (**Figures 3A**; **S3**). We performed PCR genotyping to test the integration and retention of the construct DNA in the ptDNA of plants grown in soil. Sixty-eight of the transformants had construct DNA integrated into both homology arms and 13 had construct DNA in one arm (**Figure 3B**). This suggests that the integrated markers were still retained in the ptDNA of tobacco transformants after 4 months of cultivation. Most of the tobacco transformants flowered normally and set seed with more than 80% germination rate (**Figure S3**).

For rice, since Sp did not effectively inhibit shoot regeneration, we selected transplastomic plants using Str, which effectively inhibited shoot regeneration at a concentration of 200 mg/L (**Figure S4**). Fifteen shoots were regenerated from 540 calli under Str selection. Genotyping showed integration of construct DNA into ptDNA, and three transformants had construct DNA in both homology arms under non-selective conditions (**Figure 3C**; **Table S1**). This suggests approximately not all but a part of the rice transformants maintained the integrated DNA in ptDNA. These rice transformants grew normally even in the selection medium; they also flowered and set seed with more than 80% germination rate after transferring to soil (**Figure S5**). For tobacco and rice, we confirmed the products with predicted sizes to be derived from recombination between ptDNA and the construct DNA. The remaining multiple PCR products may be derived from non-specific amplification or unintended integration of the construct DNA into ptDNA.

For kenaf, Sp selection resulted in regeneration of three shoots from ~7900 infiltrated cotyledon pieces (#1–3). We also tested infiltration of the peptide–construct DNA complex using 1000 calli induced from cotyledons and obtained one shoot (#4-C). The kenaf transformants obtained from cotyledons exhibited a partial albino phenotype 4 months after their introduction (**Figure S6**). Therefore, we genotyped several leaves from each transformant to test integration of the construct DNA into ptDNA. All the transformants showed integration of construct DNA into at least one homology arm, and most showed integration into both arms (**Figure 3D**). However, not all the leaves of each kenaf transformant had construct DNA integrated into their ptDNA. These results suggest that kenaf transformants retained the integrated pDNA in ptDNA chimerically. The kenaf transformants set seed normally (**Figure S6**).

Genotyping of ptDNA showed transformants obtained by the peptide-mediated method retained the integrated pDNA with high proportion in each plant species, and transformants sometimes showed integration of construct DNA *via* only one homology arm (**Figure 3**). Another homology arm may be integrated into an unspecified loci of ptDNA *via* microhomology-mediated recombination, which is also active in plastids in addition to the homologous recombination pathway (Kwon et al., 2010).

### Inheritance of the integrated DNA in the next generation

To test the stability of the transmission of exogenous DNA into the ptDNA of tobacco, rice, and kenaf integrated by the peptide method, we genotyped T_1_ offspring of the transformants. We cultivated T_1_ offspring of each plant under appropriate selection conditions, 500 mg/L Sp for tobacco, 100 mg/L Str for rice, and 100 mg/L Sp for kenaf, and then analyzed their genotypes. Tobacco and rice T_1_ offspring grew normally under the selection condition, while some kenaf T_1_ offspring had partially albino leaves, similar to T_0_ plants (**Figure 4A**). According to PCR genotyping, 14 of the 144 tobacco T_1_ offspring showed integration of construct DNA *via* both homology arms and 41 showed integration for one homology arm (**Figure 4B**; **Table S2**). For rice, 4 and 21 of the 180 T_1_ offspring tested had construct DNA integrated in both or one arm, respectively (**Figure 4C**; **Table S3**). Multiple PCR products in the T1 seedlings may be derived from non-specific PCR amplification since such products were frequently observed in seedlings lacking the predicted PCR products. For kenaf, one of the five T_1_ offspring tested had construct integrated DNA into both arms (**Figure 4D**). These genotyping results suggest that some offspring retained exogeneous DNA in their ptDNA. We tried to detect the ptDNA harboring the exogeneous DNA by PCR with primers amplifying the targeting plastid locus, however, such ptDNA was hardly detectable in each species (**Figure S7**), suggesting the exogeneous DNA exist in a small proportion of ptDNA in the cells. We then further carried out a DNA gel blot analysis of tobacco ptDNA to confirm the integration of exogeneous DNA in the T_1_ offspring ptDNA, using a probe that hybridizes to the ptDNA target region. Genomic DNA was digested with SmaI that cut inside and outside of the construct to show plastid targeting (**Figure S8**). The blot showed a discrete 3.2-kb band corresponding to integration of the plasmid DNA into the target site in the T_1_ offspring but not in the wild type (**Figure 4E**; **S8**). Although the intensities of the 3.2-kb band against those of the WT 11.1-kb suggest most of the plastids do not contain the integrated DNA, the blot confirms that the foreign DNA was integrated and retained in the tobacco T_1_ offspring ptDNA. On the other hand, substantial number of offspring showed resistance to Sp/Str despite lacking the construct DNA in their ptDNA. This may be because of integration of the marker gene in the parents’ nuclear DNA in addition to the ptDNA.

Finally, we observed GFP, which is encoded by the integrated DNA, in the tobacco T_1_ seedlings. Confocal laser-scanning microcopy (CLSM) showed no detectable GFP fluorescence in the WT leaf cells (**Figure 4F**), by virtue of elimination of background signal from chlorophyll autofluorescence by applying time gating (Kodama, 2016). In the same condition, we observed GFP fluorescence not homogenously but in a part of leaves of the tobacco T1 seedlings, and the GFP fluorescence exclusively localized in plastids (**Figure 4F**). This shows production of GFP from the DNA integrated and maintained in the plastid DNA. In summary, these results show integration and transmission of foreign DNA in ptDNA mediated by the fusion-peptide method.

## Conclusion

Here, we established a stable plastid transformation method using peptide-mediated gene delivery and Sp/Str selection. Foreign DNA was successfully integrated into ptDNA, maintained over long-term cultivation even without selection pressure, and transmitted to the next generation, while the transformants possess foreign DNA in ptDNA and maybe in nuclear DNA. Recent advances in nanomaterial-based methods have enabled the delivery of biomacromolecules to plant cells (Torney et al., 2007; Martin-Ortigosa et al., 2014; Mitter et al., 2017; Demirer et al., 2019; Demirer et al., 2020). For example, chitosan-complexed single-walled carbon nanotubes enabled gene delivery to chloroplasts (Kwak et al., 2019); however, this method is only suitable for transient gene delivery. The method used here is an important first step for stable plastid transformation with a biocompatible peptide carrier. Moreover, we achieved stable plastid transformation in three diverse plant species, including two dicots and one monocot: tobacco, rice, and kenaf. For kenaf, to our knowledge, this is the first report of plastid transformation. Therefore, compared to other methods, this method is easier and potentially suited to wider variety of plants. The peptide method does not require any special instruments, such as biolistic guns or electroporators. However, transformants obtained by the peptide method were not homoplasmic despite Sp/Str selection and transmission to the next generation even in tobacco, in which homoplasmic plastid transformation is well established (Maliga and Tungsuchat-Huang, 2014). This may be attributed to the efficiency and/or manner of the DNA delivery of the peptide method and could limit the potential of peptide-mediated plastid transformation. Moreover, the integrated foreign genes in such a non-homoplasmic transformants should be unstable and be lost within few generations. Increasing the delivery efficiency by using an improved peptide or cell wall-loosening reagents (Miyamoto et al., 2022) in combination with a more efficient tissue culture and selection system would enable homoplasmic plastid transformation by fusion peptides.

## Data availability statement

The original contributions presented in the study are included in the article/**Supplementary Material**. Further inquiries can be directed to the corresponding authors.

## Author contributions

MO, YH, JI, and KW performed research. MO, YH, JI, and KN analyzed data. MO and KN wrote the manuscript. All authors contributed to the article and approved the submitted version.

## Funding

This work was supported by Japan Science and Technology Agency Exploratory Research for Advanced Technology (JST-ERATO; KN, JPMJER1602), JST COI-NEXT (KN), and the Japan Society for the Promotion for Scientific Research (JSPS-KAKENHI; MO, 19K22405 and 22K05625).

## Acknowledgments

We are grateful to Drs. Shiina and Terachi for providing the plastid transformation plasmid for tobacco and to the Support Unit for Bio-Material Analysis, RIKEN CBS Research Resources Division for technical support.

## Conflict of interest

The authors declare that the research was conducted in the absence of any commercial or financial relationships that could be construed as a potential conflict of interest.

## Publisher’s note

All claims expressed in this article are solely those of the authors and do not necessarily represent those of their affiliated organizations, or those of the publisher, the editors and the reviewers. Any product that may be evaluated in this article, or claim that may be made by its manufacturer, is not guaranteed or endorsed by the publisher.
