# Supplementary material for: Functional peptide-mediated plastid transformation in tobacco, rice, and kenaf
Source: Front Plant Sci. 2022 Sep 23;13:989310. doi: 10.3389/fpls.2022.989310 (PMC9539840; doi:10.3389/fpls.2022.989310)
Supplement: Supplementary file 1 [file DataSheet_1.pdf]

Supplementary Information

Functional peptide-mediated plastid transformation in tobacco, rice, and kenaf

Masaki Odahara, Yoko Horii, Jun Itami, Kenta Watanabe, and Keiji Numata

Supplementary Figure 1-8
Supplementary Table 1-4

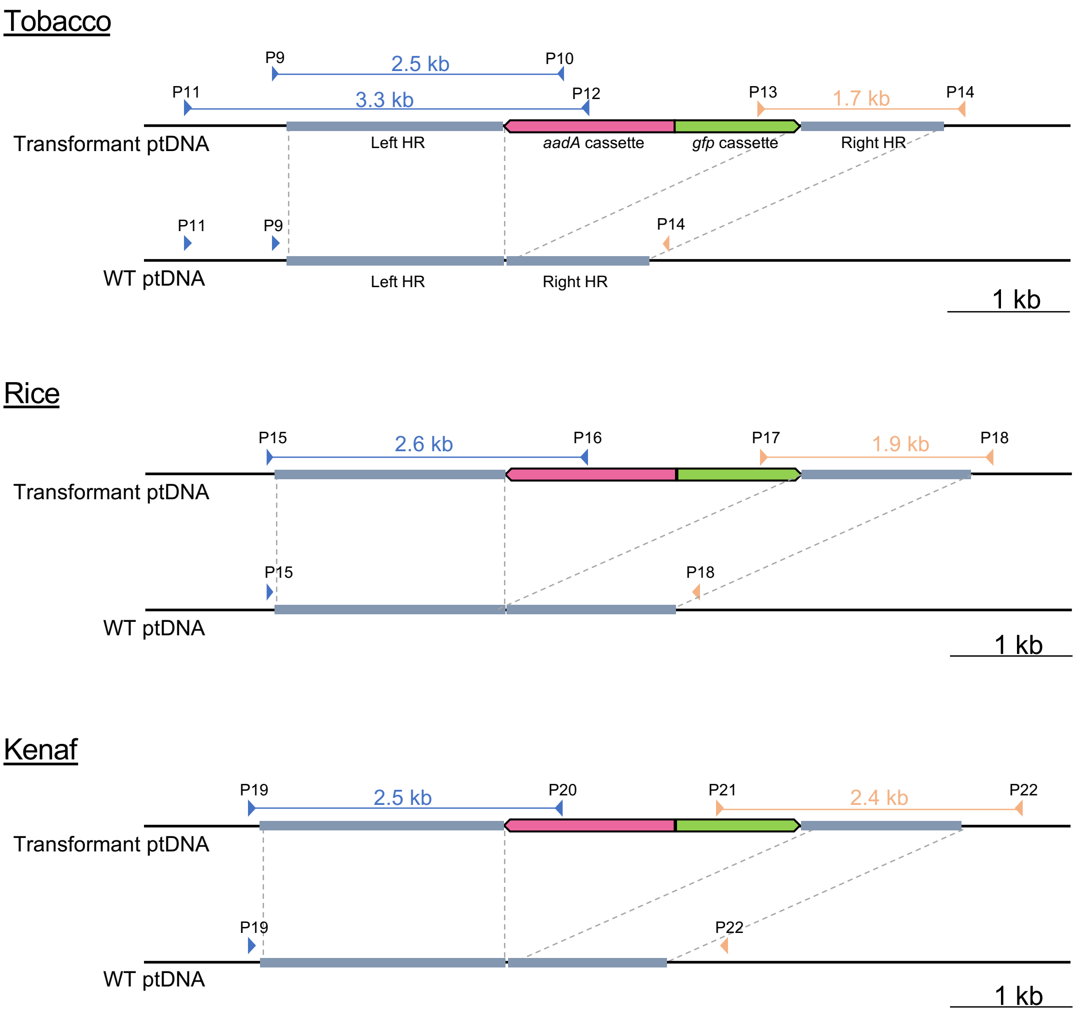

Supplemental Figure 1. Positions of primers used in genotyping

Positions of primers used in genotyping are schematically represented on the maps of transformant and WT plastid DNA. Primers used to test integration into the left and right arms are shown by blue and orange arrowheads, respectively. Since the *aadA* and *gfp* cassettes are absent in the WT, specific amplification should not occur.

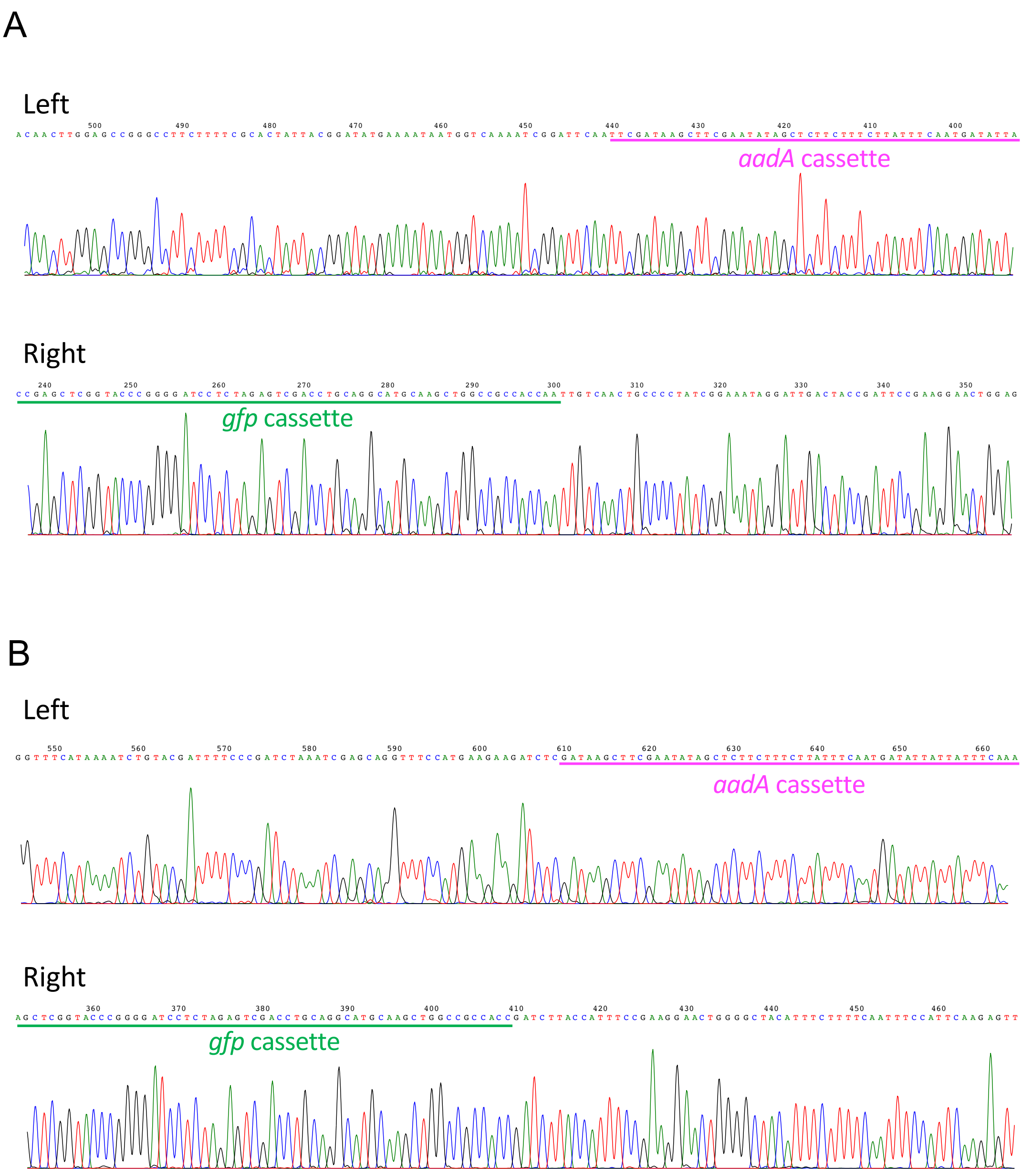

Supplemental Figure 2. Sequencing of the genotyping PCR products

A. Sequencing chromatogram of the left and right junction region between tobacco ptDNA and the construct DNA (*aadA* or *gfp* cassettes).

B. Sequencing chromatogram of the left and right junction region between rice ptDNA and the construct DNA (*aadA* or *gfp* cassettes).

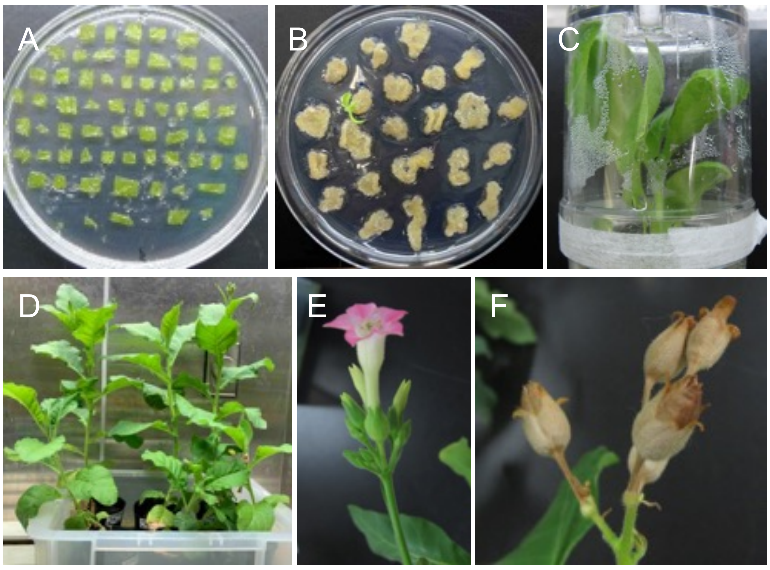

Supplemental Figure 3. Regeneration of Tobacco shoots under selection condition

A. Leaf explants transferred to a selection medium

B. Leaf explants cultivated on a selection medium for 8 weeks

C. Regenerated shoots were transferred to a medium in pot (4 months)

D. 8 months-old plants

E. and **F**. Flowering and seed setting of the transformants.

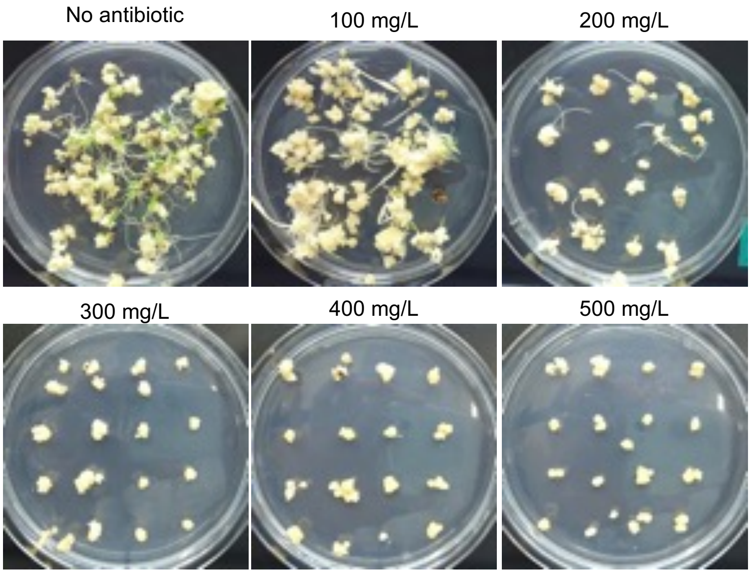

Supplemental Figure 4. Optimization of streptomycin concentration for selection of rice plastid transformants.
Rice calli induced from germinated seeds were subjected for shoot regeneration under several concentration of streptomycin.

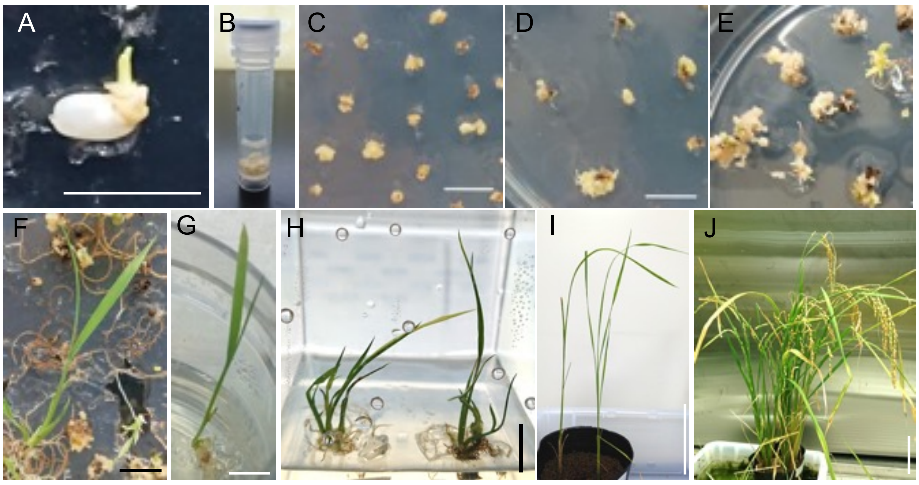

Supplemental Figure 5. Regeneration of rice plastid transformants.

A. Induction of callus from germinated rice seed.

B. Infiltration of DNA-peptide complex solution into calli.

C-**G**. Cultivation of DNA-peptide complex-treated calli on shoot regeneration medium containing streptomycin for 1 week (c), 3 weeks (d), 5 weeks (e), and 8 weeks (f and g).

H. Shoots cultivated on rooting medium.

I. Rice plastid transformants (DP5) cultivated for 11 weeks.

J. Seed setting of transformants.

Bars, 1 cm (a, c, d, f and g) and 10 cm (i and j).

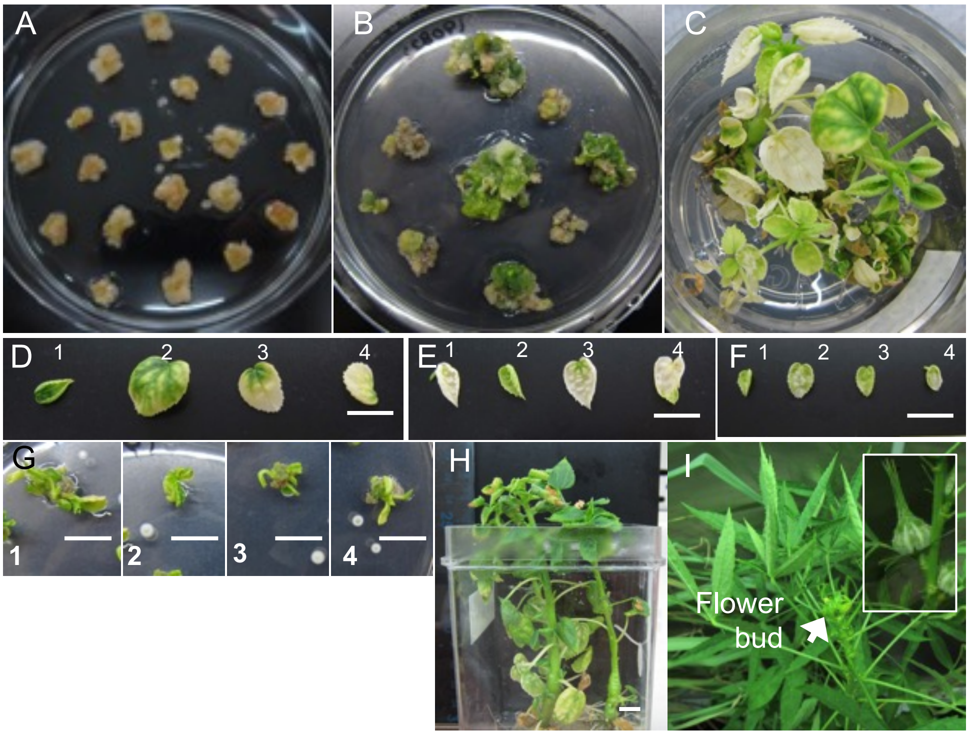

Supplemental Figure 6. Regeneration of kenaf plastid transformants under selection condition.

A. Calli induced from the infiltrated leaf explants.

B. 3 months after infiltration cultivated under selection condition.

C. 4 months after infiltration cultivated under selection condition.

D-**F**. Leaves of #1 (d), #2 (e), and #3 (f) of plants 4 months after introduction.

G. Shoots from #4-C callus

H. 5 months after infiltration cultivated under selection condition.

I. 10 months after infiltration. Transformants set flower buds indicated by an arrow and subsequent seeds shown as a flamed photo.

Bars, 1cm.

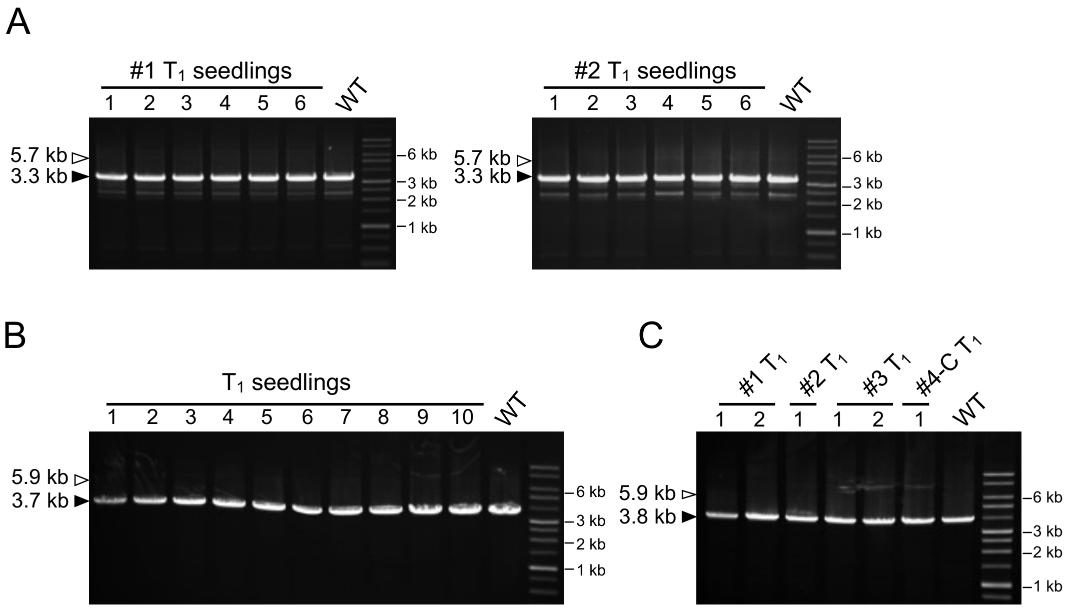

Supplemental Figure 7. Genotyping of T_1 transformants.

PCR genotyping of tobacco (A), rice (B), and kenaf (C) plastid transformants (T_1) using primers amplifying the plastid targeting loci of each plant species. Primers P9 and P14, P15 and P18, and P19 and P22 (see Fig. S1) were used in A, B, and C, respectively. Filled and blank triangles denote positions for WT ptDNA and transformant ptDNA, respectively.

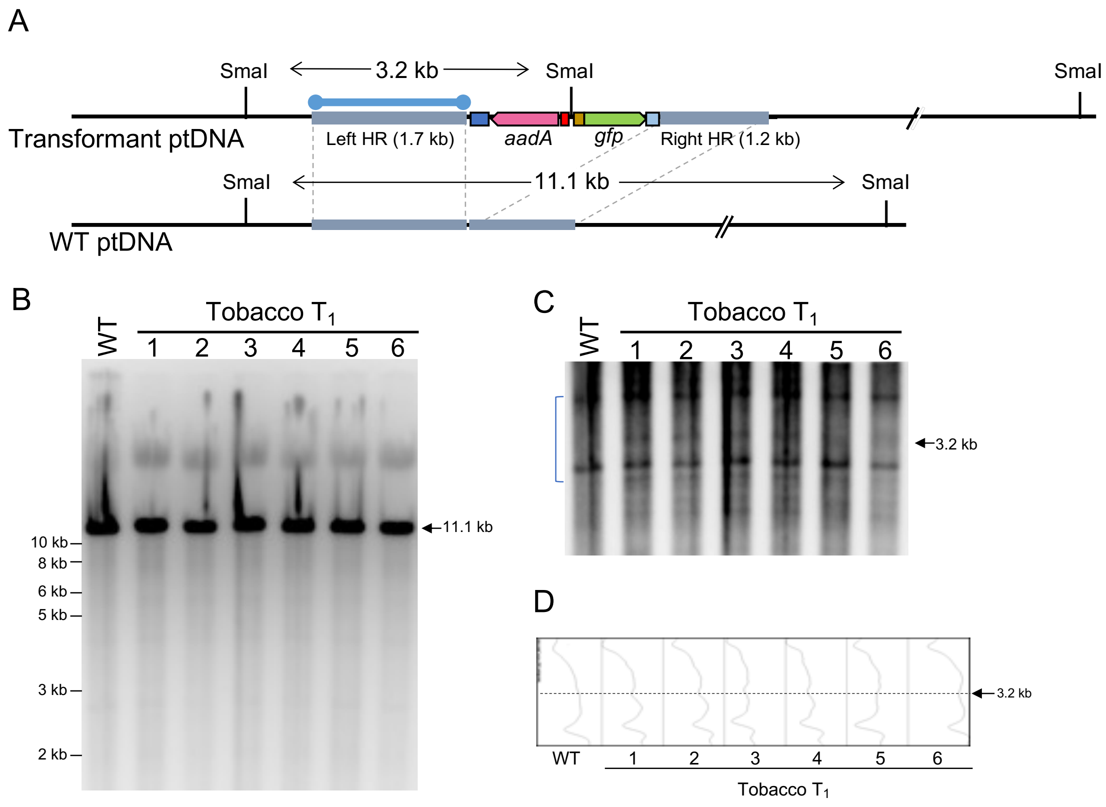

Supplemental Figure 8. DNA gel blot of tobacco T_1 transformants.

A. Maps of transformant and wild type plastid DNA targeting loci. The 1.7 kb probe corresponding to the left HR used in Figure 4e and S8B and C is shown by a bold blue line. Digestion of plastid DNA with SmaI results in 3.2 kb and 11.1 kb DNA for transformant and WT plastid DNA, respectively.

B. Gel blot of WT and tobacco T_1 transformant plastid DNA. Total genomic DNA digested with SmaI was hybridized with a probe shown in Figure S8A.

C. Long exposure of lower part of blot in (B). Band intensities of regions shown by blue line were analyzed and shown in (D).

D. Band intensities of each lane in the blot (C). Position of 3.2 kb DNA is shown by an arrow.

Supplementary Table 1. PCR genotyping result of rice transformants (T0)

	left	right
#1	+	+
#2	-	-
#3	-	-
#4	-	-
#5	+	+
#6	-	+
#7	-	-
#8	+	-
#9	-	+
#10	-	-
#11	+	-
#12	-	-
#13	-	-
#14	-	-
#15	+	+

Presence and absence of the PCR products are shown by + and -, respectively.

Supplementary Table 2. PCR genotyping result of tobacco transformants (T1)

Strain	No.	left	right	Strain	No.	left	right
#Nt_0-1A	1	+	+	#Nt_O-1 B	1	+	+
	2	+			4		+
	3	+	+		5		+
	6	+			6	+	+
	7	+			8	+	
	8		+	#Nt_O-2 D	5		+
#Nt_O-3 C	1		+	#Nt_O-3 B	1		+
	3	+	+		7	+	+
	7	+		#Nt_O-3 G	2		+
#Nt_O-4 C	1		+		7	+	+
	2		+	#Nt_O-4 A	2		+
	3		+		5	+	
	5	+	+	#Nt_O-5 F	1	+	
	7	+	+		3		+
#Nt_O-6 A	1	+	+		7		+
	3	+	+		8	+	
	6	+		#Nt_O-1 C	1		
	7	+	+		2		+
#Nt_O-7 E	1	+			6		+
	3		+	#Nt_O-1 D	7		+
	4	+	+	#Nt_O-3 A	1		+
	5	+			5		+
	6	+	+	#Nt_O-4 B	3	+	
#Nt_O-8 A	3		+		4		+
	4		+	#Nt_O-6 E	3		+
	5	+			4	+	
	8		+	#Nt_O-7 B	6		+
					8	+	

Presence and absence of the PCR products are shown by + and -, respectively.

Supplementary Table 3. PCR genotyping result of rice transformants (T1)

Strain	No.	left	right	Strain	No.	left	right
#34.1.1-2	2	+	-	#34.5.2-1	1	+	-
#34.1.1-8	8	-	+	#34.5.2-4	4	-	+
#34.1.1-10	10	+	-	#34.5.2-6	6	+	+
#34.1.1-15	15	+	+	#34.5.2-11	11	-	+
#34.1.1-23	23	+	-	#34.5.2-12	12	+	-
#34.1.1-25	25	+	-	#34.5.2-13	13	-	+
#34.1.1-26	26	-	+	#34.5.2-17	17	+	-
#34.1.1-27	27	-	+	#34.5.2-18	18	-	+
#34.1.1-28	28	-	+	#34.5.2-19	19	+	-
				#34.5.2-20	20	+	+
				#34.5.2-21	21	+	+
				#34.5.2-22	22	-	+
				#34.5.2-23	23	-	+
				#34.5.2-24	24	-	+
				#34.5.2-25	25	+	-
				#34.5.2-26	26	+	-

Presence and absence of the PCR products are shown by + and -, respectively.

Supplementary Table 4. Oligo DNA primers used in this study

Name	Sequence (5'->3')
P1	CGGTACCCGGGGATCGGGAACGGATTCACCGCC
P2	CGACTCTAGAGGATCGTAAGGCAGAGTTGGGTTT
P3	TCCATGAAGAAGATCTCGATAAGCTTCGAATATAGC
P4	GGAAATGGTAAGATCGGTGGCGGCCAGCTTGCA
P5	CGGTACCCGGGGATCGGGAACGAATTCACCGCC
P6	CGACTCTAGAGGATCGATCTTTCTCGATCAATCCC
P7	TCTTCTTGCGCCAAACGATAAGCTTCGAATATAGC
P8	GGAAATGGTAAGATCAATTGGTGGCGGCCAGCTTG
P9	CCAGTACGGCTACCTTGTTACGAC
P10	CGTTGTCCCGCATTTGGTAC
P11	GCTCCATTTTATTTTCCCATTGCTAAACC
P12	CTGGCGATGAGCGAAATGTAGTGC
P13	TCTGCCCTTTCGAAAGATCCC
P14	CCCGAAGAGTAACTAGGACCAATTTAGTC
P15	CAGTCGCAAGCCTAGCCTTA
P16	CGTTGTCCCGCATTTGGTAC
P17	TCTGCCCTTTCGAAAGATCCC
P18	TAGGGCTCCTCGAATAATGCG
P19	TGCGGTTAAGGTAACGACTTC
P20	ACTACGTGAAAGGCGAGATCA
P21	CAAGACACGTGCTGAAGTCAA
P22	TAGGGTTCCTCGAACAATGTG
P23	TAGCGATTCCGGCTTCATGC
P24	TGAGTTTCTCGACCCTTTGAC
